# Sialylation in Colorectal Cancer Rewires Antitumor Immunity at the Peritoneal Metastatic Site

**DOI:** 10.1002/eji.70204

**Published:** 2026-05-19

**Authors:** Irene van der Haar Àvila, Kristiaan Lenos, Victor Lorrain, Eleonora Nardini, Ernesto Rodríguez, Juan J. García‐Vallejo, Yvette van Kooyk, Joep Grootjans, Sandra J. van Vliet

**Affiliations:** ^1^ Amsterdam UMC Location Vrije Universiteit Amsterdam Department of Molecular Cell Biology and Immunology Amsterdam the Netherlands; ^2^ Cancer Center Amsterdam Cancer Biology and Immunology Amsterdam the Netherlands; ^3^ Amsterdam Institute for Immunology and Infectious Diseases Cancer Immunology Amsterdam the Netherlands; ^4^ Center for Experimental and Molecular Medicine Laboratory for Experimental Oncology and Radiobiology Amsterdam UMC Location Vrije Universiteit Amsterdam Amsterdam the Netherlands; ^5^ Oncode Institute Amsterdam the Netherlands; ^6^ Department of Gastroenterology and Hepatology Amsterdam UMC, Location Vrije Universiteit Amsterdam Amsterdam the Netherlands; ^7^ Amsterdam Gastroenterology Endocrinology Metabolism Amsterdam the Netherlands

**Keywords:** colorectal cancer, peritoneal metastasis, sialic acids, tumor immunity

## Abstract

Colorectal cancer (CRC) patients are frequently diagnosed with metastases to the peritoneal cavity. These patients have a dismal prognosis and only limited therapeutical benefits. Solid tumors, including CRC, often display elevated levels of sialylated glycan structures, acting as immune checkpoints for immune evasion. However, the detrimental effects of tumor sialylation on antitumor immunity at the peritoneal site remain unexplored. We uncovered that sialylation is higher in peritoneal metastases (PM) compared with primary CRC or liver metastases. Intraperitoneal injection of sialic acid‐deficient CT26 CRC cells led to significantly less peritoneal tumor development and a lower peritoneal cancer index (PCI) score. Sialic acid‐devoid tumors harbored higher numbers of live CD45^+^ cells, and the myeloid compartment of desialylated tumors showed increased levels of MHC‐II^+^ macrophages and dendritic cells and significantly fewer neutrophils and CD206^+^ macrophages, correlating with a lower PCI score. In conclusion, the absence of sialylated glycans reprograms antitumor immunity and reduces tumor formation at the peritoneal site.

## Graphical abstract

This study shows that loss of sialylation in CT26 cells reduces peritoneal tumor burden, correlating with a lower peritoneal cancer index score. These tumors exhibited increased immune infiltration with prominent alterations in the myeloid compartment and immune cell correlations unique to desialylated tumors, indicating profound reshaping of antitumor immunity.

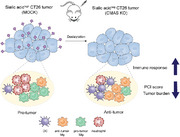

**Abbreviations**
7AAD: 7‐aminoactinomycin
BSA: bovine serum albumin
cDC1: conventional type 1 dendritic cell
CMAS: cytidine monophosphate *N*‐acetylneuraminic acid synthetase
CMS: consensus molecular subtype
CRC: colorectal cancer
CTLA‐4: cytotoxic T‐lymphocyte‐associated protein 4
DC: dendritic cell
ECM: extracellular matrix
gRNA: guideRNA
KO: knockout
LM: liver metastases
MDSC: myeloid‐derived suppressor cell
MSS: microsatellite‐stable
NEU: neuraminidase
Neu5Ac: *N*‐acetylneuraminic acid
NK: natural killer
NS: not significant
PCI: peritoneal cancer index
PD‐1: programmed cell death protein 1
pDC: plasmacytoid dendritic cell
PM: peritoneal metastases
Sia: sialic acid
Siglec: sialic acid‐binding immunoglobulin‐like lectin
TAM: tumor‐associated macrophages
TAN: tumor‐associated neutrophil
TME: tumor microenvironment
Treg: regulatory T cell
Trm: tissue‐resident memory

## 1 Introduction

Colorectal cancer (CRC) is among the top three causes of cancer‐related deaths. Patients with metastases to the peritoneal cavity have the worst prognosis with a median survival of only 11 months [1, 2]. Peritoneal metastases (PMs) are frequently detected at a late stage when treatment options are scarce, and response to systemic therapy is limited [3]. CRC‐PMs are mostly microsatellite‐stable (MSS) tumors derived from the mesenchymal consensus molecular subtype (CMS) 4, distinguished by increased levels of TGF‐β, immune suppression, and stromal invasion [4, 5, 6]. Recent evidence suggests that the immune landscape in CRC‐PMs differs significantly from that of the primary tumor, indicating it should be treated as a distinct disease [7, 8]. Therefore, to improve therapeutic options for these patients, a better understanding of the peritoneal tumor microenvironment (TME) is required.

During the past years, it has become evident that tumor cells display aberrant glycans to avoid immune recognition. Upregulation of sialylated glycans on cancer cells, as well as cancer‐associated fibroblasts and endothelial cells [9, 10], contributes to the formation of an immunosuppressive milieu and dampens the antitumor immune response by engaging inhibitory receptors called Sialic acid (Sia)‐binding immunoglobulin‐like lectins (Siglecs) [11]. Binding of Sias to specific Siglec receptors suppresses NK cell cytotoxicity [12, 13, 14] and CD8^+^ T cell responses [15]. Dendritic cells (DCs) can adopt a tolerogenic phenotype upon binding sialoglycans, limiting T cell priming and stimulating regulatory T cell (Treg) generation [16, 17, 18]. In macrophages, Siglec engagement reduces their phagocytic capacity [19] and favors polarization toward an immunosuppressive M2‐like phenotype [20, 21]. Elimination of tumor sialylation results in decreased tumor growth in vivo, associated with increased CD8^+^ T cell and reduced Treg infiltration in melanoma, breast cancer, and pancreatic cancer [22, 23, 24, 25]. In addition to instigating immune evasion, Sias regulate tumor proliferation, invasion, and migration [26, 27].

We have previously demonstrated that complete removal of Sias on murine CT26 CRC cells led to a severely diminished subcutaneous tumor growth, associated with an increase in cytotoxic NK cells and γδ T cells [28]. How sialylation modulates the TME at the peritoneal site has never been explored. In this study, we employed the peritoneal metastasis model described by Bastiaenen et al. [29], which recapitulates the heterogeneity observed in CRC patients. We discovered that Sias strongly impact CRC‐PM, with fewer peritoneal tumors developing when Sias are absent. Sia‐deficient tumors displayed a more immunogenic TME characterized by an increase in DCs and antigen‐presenting cells expressing MHC‐II, and a reduction in neutrophils. Analysis of human CRC samples revealed enhanced expression of sialylation‐related genes in PMs compared to the primary tumors, indicating that the peritoneal cavity likely exhibits a more immunosuppressive milieu, capable of dampening the antitumor immune response.

## 2 Results

### 2.1 Increased Sialylation Gene Profiles in Human CRC Peritoneal Metastasis

**FIGURE 1:**
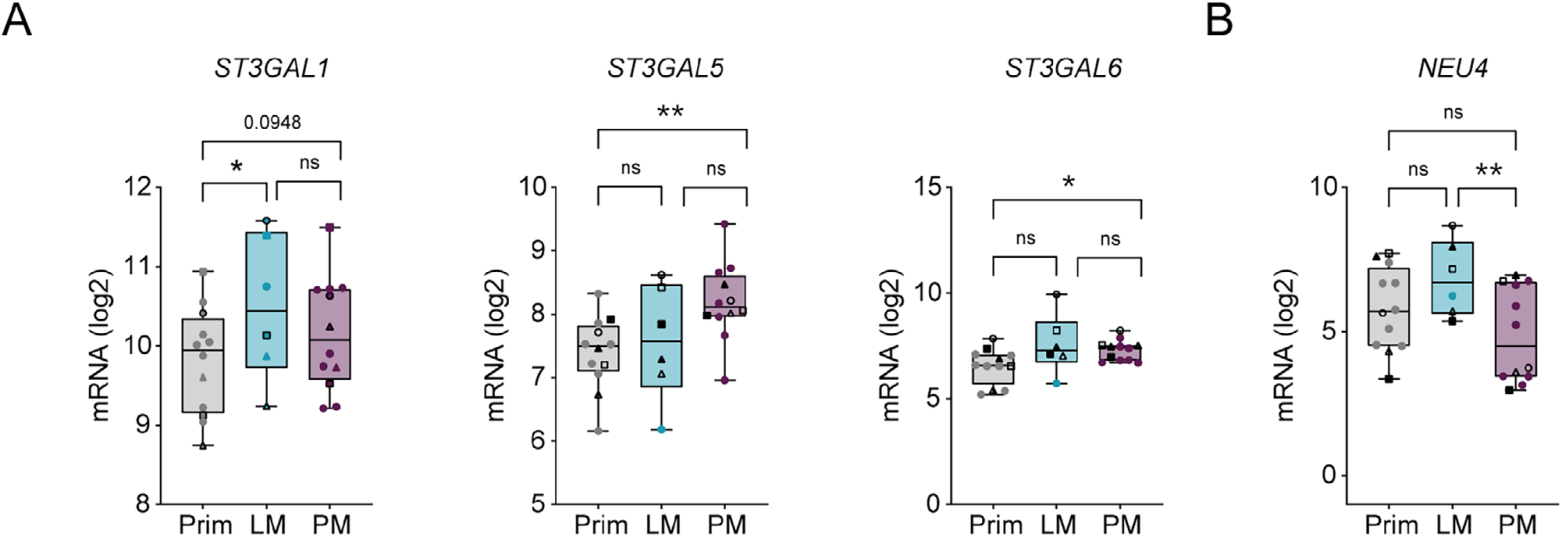
Expression of sialylation‐related genes in CRC primary tumor (Prim), liver metastasis (LM) and peritoneal metastasis (PM). Boxplots showing mRNA expression of sialyltransferases (A), neuraminidase 4 (*NEU4*) (B) in primary tumor and liver or peritoneal metastases. Data are shown as mean ± SD; pairwise comparisons with a mixed linear effects model (*p ≤ 0.05; **p ≤ 0.01). (Prim, *n* = 12 patients, 35 samples; LM, *n* = 6 patients, 6 samples; and PM, *n* = 12 patients, 59 samples).

To analyze whether expression of sialylation‐related genes differs between anatomical locations, we analyzed bulk RNA sequencing data from matched primary CRC, liver metastases (LMs), and PM in 100 samples from 12 patients with CMS4 subtype CRC. We identified significantly higher expression of the sialyltransferases *ST3GAL5* and *ST3GAL6*, involved in the synthesis of α2‐3‐linked Sias, in PMs compared with primary tumors (Figure 1A). Only with the *ST3GAL1* gene did we observe a significant difference between primary tumors and LMs. Gene expression of the sialidase *NEU4*, an enzyme that degrades sialylated structures, was significantly lower in PMs compared with LMs, and slightly reduced compared with primary tumors (Figure 1B). Together, this suggests a “glyco”‐reshaping of the TME in PMs toward an altered sialylation profile, in particular of α2‐3‐linked Sia. Other sialyltransferases, Siglecs, and neuraminidase genes were not significantly altered.

### 2.2 Loss of Sia Reduces Metastases Formation In Vivo

**FIGURE 2:**
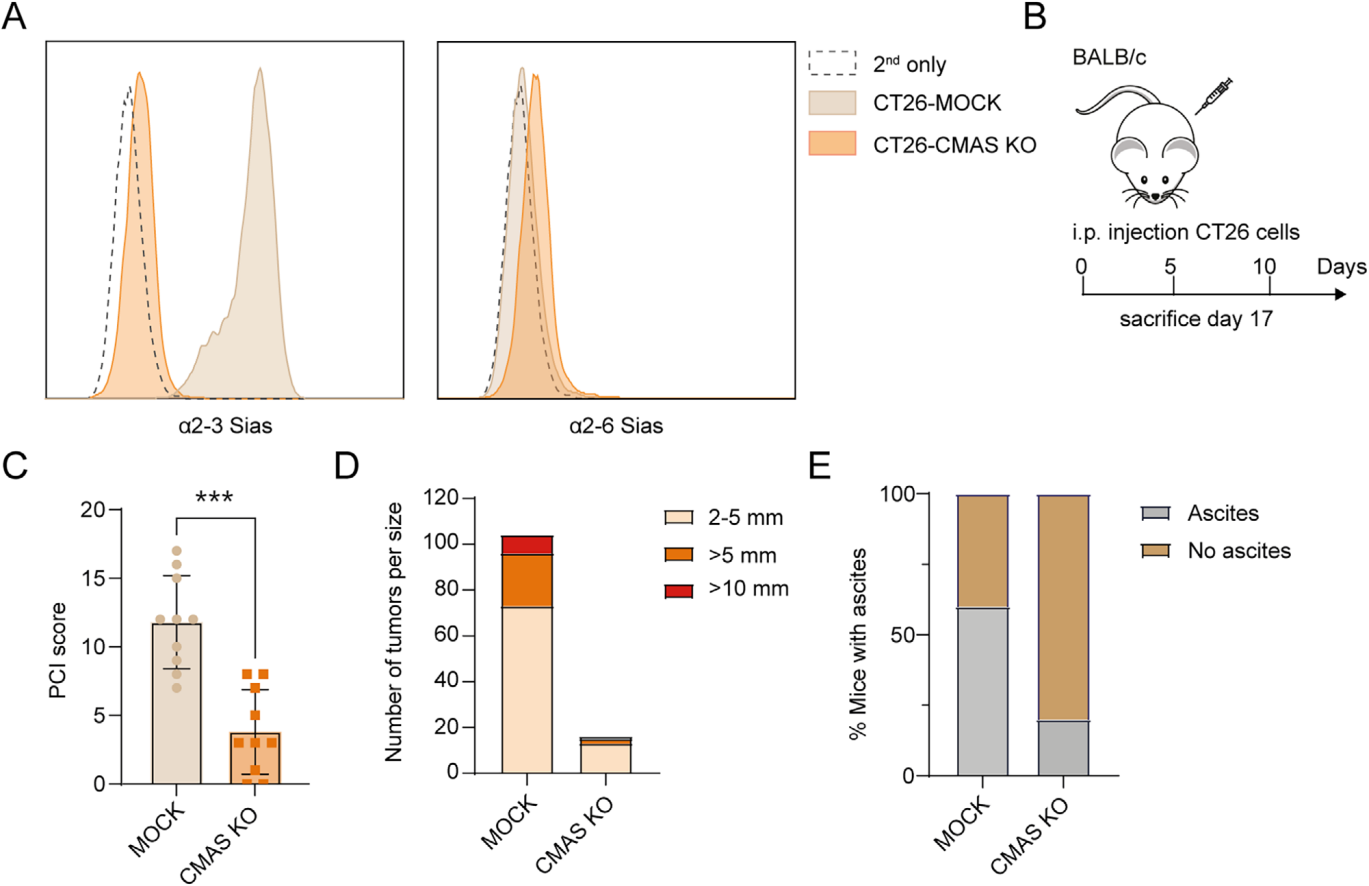
Loss of Sias results in diminished peritoneal tumor growth in a CT26 mouse model. (A) The presence of α2‐3 and α2‐6 Sias in CT26‐MOCK and CMAS KO cells was assessed by flow cytometry using α2‐3 and α2‐6 Lectenz, respectively. (**B**) CT26‐MOCK or CT26‐CMAS KO cells were injected intraperitoneally (i.p.) into BALB/c mice (*n* = 20). Mice were sacrificed 17 days posttumor inoculation, followed by dissection and evaluation of peritoneal metastases. (**C**) PCI score used to assess the extent of peritoneal metastases of CT26‐MOCK and CT26‐CMAS KO tumors. Data is shown as mean ± SD; unpaired nonparametric *t‐*test (***p ≤ 0.001). (**D**) Number of tumors classified per size between MOCK and CMAS KO‐tumor bearing mice. (**E**) Percentage of mice with ascites formation in the peritoneum.

To determine how increased tumor cell sialylation, as observed in human PM, impacts cancer growth and antitumor immunity at the peritoneal site, we employed a peritoneal metastasis model using the MSS‐like CT26 murine cell line. Similar to human PMs, CT26 cells have high expression of α2‐3 Sias (Figure 2A). Given that multiple sialyltransferases create the high α2‐3 sialylation phenotype, and deletion of one enzyme is compensated for by others, we eliminated all Sias by knocking out the *Cmas* gene, as previously described [30]. The CMAS enzyme activates the substrate required for Sia addition to glycoproteins or glycolipids. *Cmas* gene KO resulted in a complete loss of sialylation on CT26‐CMAS KO cells (Figure 2A), without affecting cell proliferation in vitro [28].

We subsequently studied tumor formation by intraperitoneal injections of CT26‐MOCK and CT26‐CMAS KO cells into BALB/c mice, as described [29] (Figure 2B). Compared with CT26‐MOCK, CT26‐CMAS KO cells showed strongly reduced peritoneal tumor formation (Figure 2C; Figure S1). Both the number and size of tumors present in the peritoneum of mice grafted with CT26‐CMAS KO were reduced, as reflected by the PCI score (Figure 2C,D). We also detected significantly more ascites in mice with CT26‐MOCK tumors (Figure 2E), a common sign of advanced disease in humans [31].

### 2.3 Enhanced Lymphoid Infiltration in Sias‐Devoid Tumors

**FIGURE 3:**
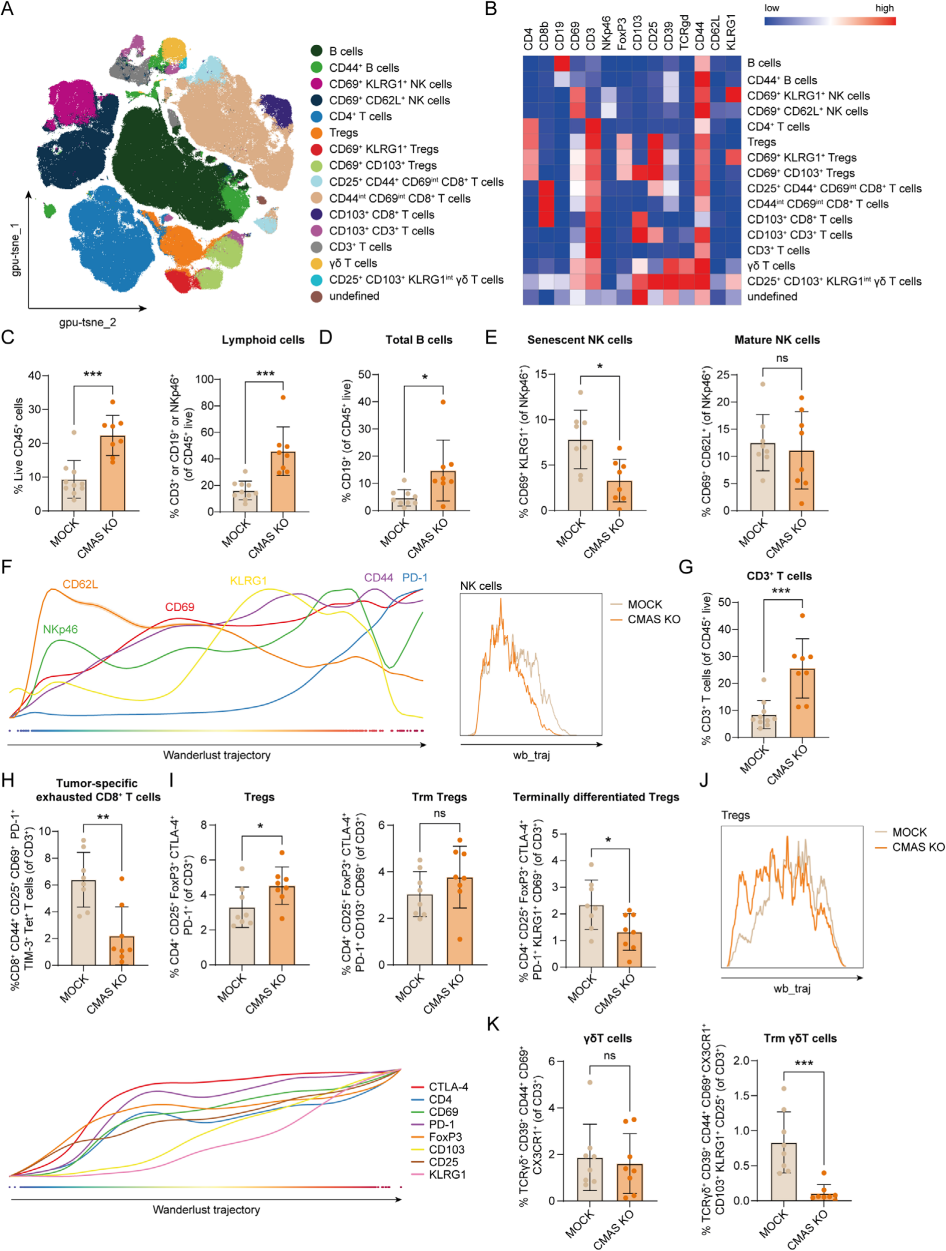
Loss of tumor sialylation alters the frequencies of lymphoid subsets in a CRC peritoneal tumor model. (A) tSNE of unsupervised clustering analysis, showing the dimensional reduction and FLOWSOM clustering of spectral flow cytometry data on alive lymphoid cells in CT26 tumors. (**B**) Heatmap displaying the median scaled intensities of all the markers across the annotated immune cell clusters shown in (**A**). (**C**) Percentage of alive CD45^+^ cells and lymphoid cells (either CD3^+^, CD19^+^, or NKp46^+^) of CT26‐MOCK and CT26‐CMAS KO tumors, respectively. (**D**) Percentage of B cells (CD19^+^) in both CT26 tumors gated over CD45^+^ alive cells. (**E**) Percentage of different NK cells subsets obtained after unsupervised clustering of the lymphoid population (NKp46^+^). (**F**) Wanderlust trajectory analysis of the different NK cell clusters in CT26‐MOCK and CT26‐CMAS KO tumors. (**G**) Percentage of CD3^+^ T cells in both CT26 tumors gated over CD45^+^ alive cells. (**H**) Percentage of tumor‐specific exhausted CD8^+^ T cells (CD8^+^ CD69^+^ CD25^+^ PD‐1^+^ TIM‐3^+^ Tet^+^) in CT26 tumors, gated over CD3^+^ T cells. (**I**) Percentage of distinct Tregs (CD4^+^ CD25^+^ FoxP3^+^ CTLA‐4^+^ PD‐1^+^) subsets obtained after FLOWSOM unsupervised clustering analysis of the lymphoid population. (**J**) Wanderlust trajectory analysis of the Tregs subsets. (**K**) Percentage of γδ T cells (TCRγδ^+^ CD39^+^ CD44^+^ CD69^+^ CX3CR1^+^) clusters identified after unsupervised clustering analysis of the lymphoid population. Each dot represents one individual mouse. Data is shown as mean ± SD; unpaired nonparametric *t‐*test (ns, not significant; *p ≤ 0.05; ***p ≤ 0.001).

Next, we analyzed immune cell infiltration in CT26‐MOCK and CT26‐CMAS KO peritoneal tumors using high‐dimensional spectral flow cytometry analysis. FLOWSOM unsupervised clustering analysis of the lymphoid compartment resulted in the identification of 16 different subsets based on lymphoid differentiation and activation markers (Figure 3A,B; Figure S2A,B). CT26‐CMAS KO peritoneal tumors harbored relatively higher levels of live CD45^+^ and lymphoid cells (Figure 3C), both negatively correlating with the PCI score (Figure S3A), indicating an immune‐driven effect. Furthermore, Sia‐devoid peritoneal tumors contained both more T and B cells, but fewer senescent KLRG1^+^ NK cells [32] (Figure 3D–G), whereas mature NK cells (CD69^+^CD62L^+^) were present in similar percentages in both tumor types (Figure 3E). We next performed trajectory analyses using the Wanderlust algorithm (Figure S3B). We observed a different trajectory state and differentiation program of NK cells in MOCK compared with CMAS KO tumors, with a shift toward a terminally differentiated phenotype in the MOCK tumors (Figure 3F). Together, these data indicate that more activated NK cells infiltrate CMAS KO tumors, potentially contributing to enhanced tumor control.

Infiltration of CD3^+^ and CD3^+^CD4^+^ T cells was significantly increased in CMAS KO tumors (Figure 3G; Figure S3C). In contrast, a cluster of exhausted CD8^+^ T cells, specific for the gp70 tumor antigen (CD8^+^CD69^+^CD44^+^CD25^+^PD‐1^+^TIM‐3^+^Tet^+^), was diminished in tumors without Sias, while tissue‐resident CD103^+^CD8^+^ T cells were increased (Figure 3H, Figure S3C). Additionally, we observed a decrease in terminally differentiated Tregs (CD69^+^KLRG1^+^), whereas CD103^−^CD69^−^KLRG1^−^ Tregs were enhanced in CMAS KO tumors (Figure 3I). All Treg subsets had high expression of CTLA‐4 and, to a lesser extent, PD‐1 (Figure S3D; Figure 3I), suggesting an activated or potentially suppressive state. Wanderlust trajectory analysis of the Treg compartment (Figure S3D) revealed clear distinctions in the differentiation state of Tregs between MOCK and CMAS KO tumors (Figure 3J). CT26 tumors harbored 2 clusters of γδ T cells, of which the CD103^+^KLRG1^+^CD25^+^ γδ T cell subset, with tissue‐resident memory (Trm) properties, was significantly decreased in CMAS KO tumors (Figure 3K). Altogether, these findings indicate a reshaping of the lymphoid compartment when tumor cells lack Sias.

### 2.4 Loss of Tumor Sialylation Reshapes the Myeloid Compartment

**FIGURE 4:**
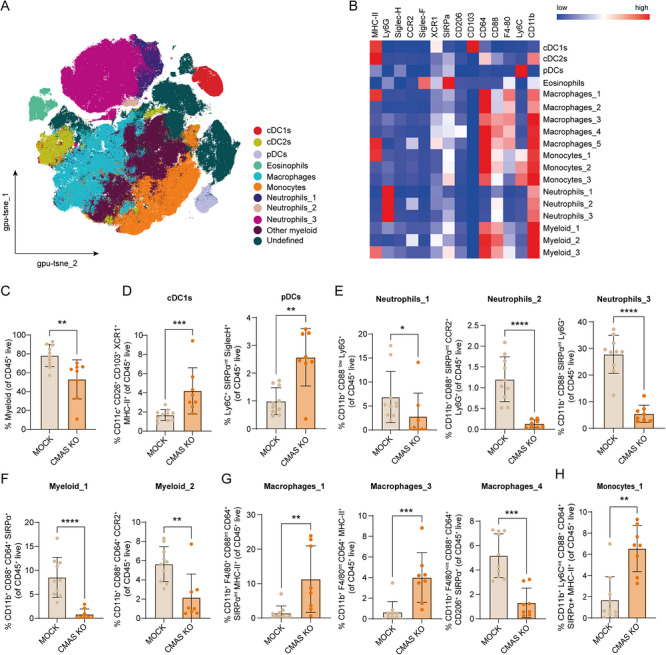
Sialic acid‐deficient tumors show a distinct myeloid cell composition compared with sialylated tumors. (A) tSNE plots of unsupervised clustering analysis, showing the dimensional reduction and PARC clustering of spectral flow cytometry data on alive myeloid cells in CT26 tumors. The macrophage and monocyte clusters are presented together, respectively, for visualization purposes. (**B**) Heatmap displaying the median scaled intensities of all the markers across the annotated immune cell clusters shown in (**A**). (**C**) Percentage of myeloid cells, gated from CD45^+^ alive cells and after excluding the lymphoid population, of CT26‐MOCK and CMAS KO tumors. (**D**) Percentage of pDC and cDC1 subsets obtained after unsupervised clustering of the myeloid compartment. (**E**) Percentages of three distinct neutrophil clusters obtained after PARC analysis, in sialylated tumors and nonsialylated tumors. (**F**) Percentage of two clusters named as myeloid and defined as CD11b^+^, Ly6C^−^, F4/80^−^, Ly6G^−^, and Siglec‐F^−^. (**G**) Percentages of three significant macrophage subsets identified after unsupervised clustering analysis. (**H**) Percentage of monocytes in CT26‐MOCK and CT26‐CMAS KO tumors, obtained after unsupervised clustering. Each dot represents one individual mouse. Data is shown as mean ± SD; unpaired nonparametric *t‐*test (ns, not significant; *p ≤ 0.05; **p ≤ 0.01; ***p ≤ 0.001; ****p ≤ 0.0001).

Unsupervised clustering of the myeloid compartment revealed 18 distinct clusters, based on the expression of myeloid differentiation and activation markers (Figure S4A,B; Figure 4A,B). Overall, CT26‐CMAS KO peritoneal tumors harbored fewer myeloid cells than MOCK tumors (Figure 4C), positively correlating with the PCI score (Figure S5A). CT26‐CMAS KO tumors contained more cDC1s and plasmacytoid DCs (pDCs) (Figure 4D), while all neutrophil populations were significantly decreased (Figure 4E). We identified a reduction of myeloid_1 (CD11b^+^CD88^+^CD64^+^SIRPα^+^) and myeloid_2 (CD11b^+^CD88^+^CD64^+^CCR2^+^) subsets (Figure 4F), whereas the MHC‐II^+^ monocytes cluster (monocytes_1) was mainly present in Sia‐devoid tumors (Figure 4G). Of the five macrophage populations, three were significantly different between CT26‐MOCK and CT26‐CMAS KO tumors. Fewer CD206^+^ macrophages, reminiscent of a M2‐like subset, were found in CT26‐CMAS KO tumors (macrophages_4, Figure 4G), while potential antigen‐presenting MHC‐II^high^ macrophages were substantially elevated (macrophages_1 and macrophages_3, Figure 4G). All other myeloid clusters can be found in Figure S5B. Based on their role in tumor immunosuppression, we also assessed expression of Siglec‐E and Siglec‐15 [15, 33]. Of all myeloid populations, macrophages and neutrophils predominantly expressed Siglec‐E, while none expressed Siglec‐15 (Figure S5C). Siglec‐E expression was reduced in the neutrophils_2 subset (Figure S5D) following tumor desialylation, while macrophage Siglec‐E expression remained unchanged (Figure S5E).

### 2.5 Immune Correlations Indicate Extensive Remodeling of the Immune TME in Sia‐Devoid Tumors

**FIGURE 5:**
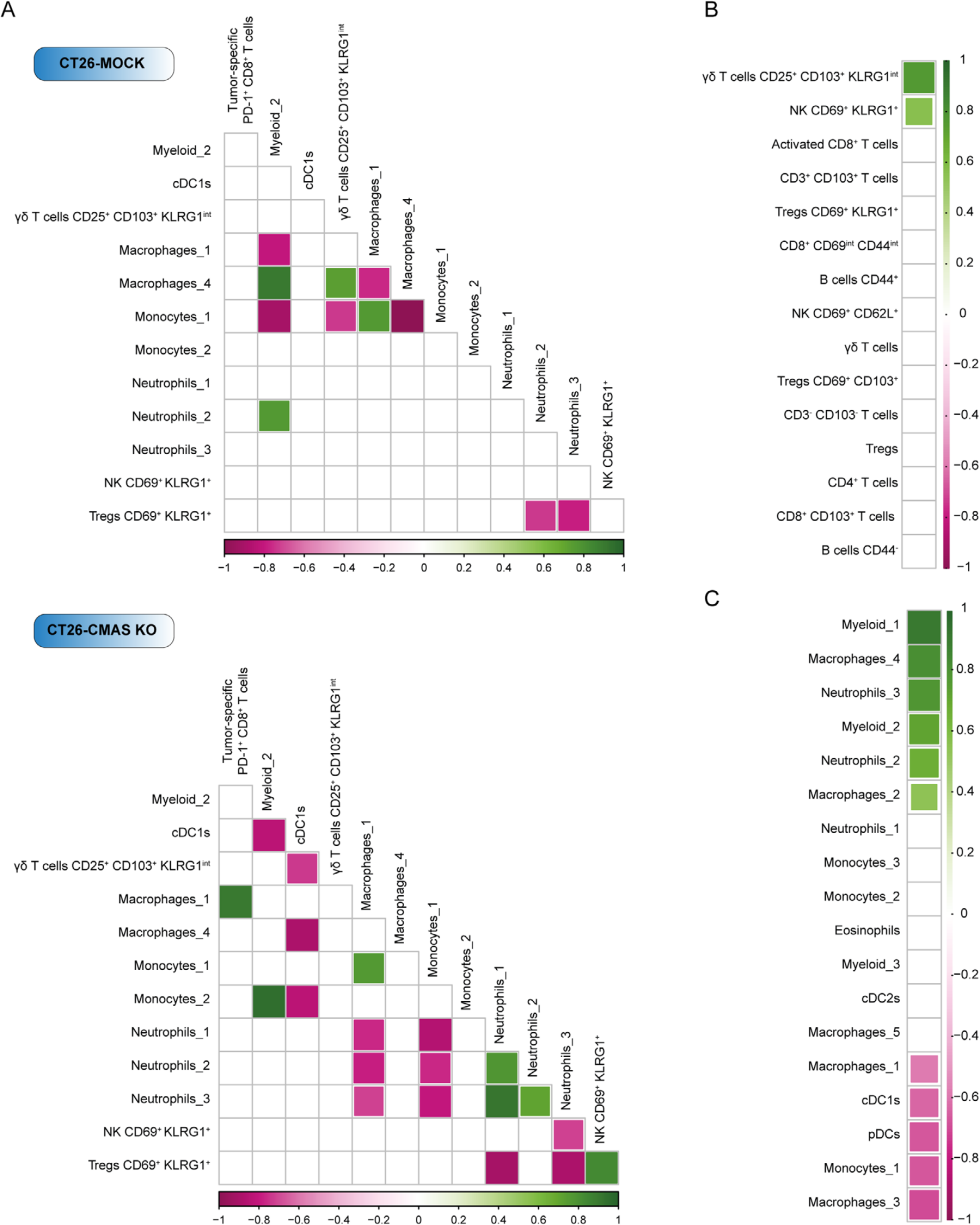
Immune subset correlations in the peritoneal TME. (A) Spearman correlation coefficients for selected lymphoid and myeloid populations were calculated in R using the corr.test function, and the results were visualized with the corrplot package (colors indicate the matched correlations between immune cell subsets that were only significant in CMAS KO tumors). (**B**) Spearman's correlations between the PCI score and the distinct lymphoid populations identified with unsupervised FLOWSOM clustering analysis. (**C**) Spearman's correlations between the PCI score and the myeloid subsets obtained with unsupervised PARC clustering analysis. Only significant correlations are shown, whereby a green color indicates a positive correlation and a pink color a negative correlation between subsets.

To assess immune cell dynamics in the TME, we performed Spearman's correlations between all lymphoid and myeloid clusters obtained with separate unsupervised clustering analysis of CT26‐MOCK and CT26‐CMAS KO tumors (Figure S6). Interestingly, patterns of immune cell subset correlations were distinct in the CT26‐MOCK TME compared with the CT26‐CMAS KO TME, suggesting that tumors devoid of Sias have a complete rewiring of immune cell networks (Figure 5A). For instance, only in CT26‐CMAS KO tumors, tumor‐specific CD8^+^ T cells positively correlated to MHC‐II^+^ macrophages (macrophages_1, macrophages_3, and macrophages_5) (Figure 5A; Figure S6). Likewise, MHC‐II^+^ macrophages_1 and monocytes_1 negatively correlated with all neutrophil clusters in CMAS KO tumors. The enhanced presence of cDC1s in CT26‐CMAS KO tumors was also paired with a decrease in CD206^+^ M2‐like macrophages (macrophages_4), monocytes_2, myeloid_2 cells, and CD103^+^KLRG1^+^ γδ T cells. Furthermore, the decline in senescent NK cells (CD69^+^KLRG1^+^) observed in Sia‐devoid tumors was positively associated with the reduction of terminally differentiated KLRG1^+^Tregs (Figure 5A; Figure S6).

Next, we conducted Spearman's correlation analyses to examine the relationship between the immune cell populations and the PCI score. KLRG1^+^CD69^+^ NK cells and CD103^+^CD25^+^KLRG1^int^ γδ T cells, both significantly downregulated in CT26‐CMAS KO tumors (Figure 3E,K), positively correlated with the PCI score (Figure 5B). Neutrophils_2, neutrophils_3, myeloid_1, myeloid_2, macrophages_4, reduced in CT26‐CMAS KO tumors (Figures 4E–G), also positively correlated with the PCI score (Figure 5C), implying that these subsets are immunosuppressive and sustain tumor growth. On the contrary, the cross‐presenting cDC1s and pDCs, together with MHC‐II^+^ macrophages and monocytes (macrophages_1, macrophages_3, and monocytes_1), were inversely associated with the PCI score (Figure 5C). These myeloid subsets were significantly increased in Sia‐lacking tumors.

In conclusion, our data show that the loss of Sias in CT26 CMAS KO peritoneal tumors results in significant alterations in immune cell frequencies, particularly within the myeloid compartment, reflecting a reprogrammed, pro‐inflammatory immune landscape that correlates with improved tumor control and a reduced PCI score.

## 3 Discussion

Many cancer types display an aberrant glycosylation profile as a mechanism of immune evasion [34, 35, 36]. Specifically, the upregulation of Sias on tumor cell surfaces and their interaction with Siglecs can dampen antitumor immunity, creating an immunosuppressive microenvironment. Yet, the exact role of sialylation in metastatic tumors in vivo remains largely unexplored. So far, only one study has shown that knocking out *Cmas* significantly decreased breast cancer metastases in an orthotopic mouse model [37].

Analysis of sialylation‐related gene expression in PMs from CRC patients revealed an increase compared with primary tumors in sialyltransferases *ST3GAL5* and *ST3GAL6*, accompanied by a downregulation of *NEU4*. NEU4 has been associated with a more invasive cancer phenotype [38], while sialyl Lewis X, synthesized by ST3GAL6, is similarly linked to malignancy [39].

We demonstrate that sialylated CT26 cells display significantly increased tumor growth, which was associated with reduced immune cell infiltration in the TME. Additionally, peritoneal CT26‐MOCK tumor formation was accompanied by ascites accumulation, which has previously been shown to contain high levels of the immunosuppressive cytokines IL‐10 and TGF‐β [40, 41]. Analysis of the immune landscapes revealed significant differences between CT26‐MOCK and CT26‐CMAS KO tumors, with the most notable alterations occurring within the myeloid compartment. This was further corroborated by significant correlations between the myeloid population and the PCI scores. Nevertheless, Sia‐devoid tumors had significantly increased lymphoid cells while the myeloid population was reduced. Interestingly, in a subcutaneous CT26 model, lymphoid and myeloid cell frequencies were comparable between MOCK and CMAS‐KO [28], confirming previous work demonstrating that the CRC tumors can exhibit distinct immune TMEs across different organ sites [8].

In CRC patients with LMs, the immune compartment was dominated by high expression of T cell exhaustion markers on both CD4^+^ and CD8^+^ T cells, indicative of a severely suppressed local immune microenvironment [7, 8, 40]. In the present study, CD8^+^ T cells from CT26 tumors exhibited a similar exhausted tumor‐reactive phenotype, indicated by the expression of CD69, PD‐1, and TIM‐3.

Intriguingly, we observed increased Treg numbers in CMAS KO tumors, despite reduced tumor growth. While Tregs are generally associated with immunosuppression, it is important to note that their prognostic value in CRC remains controversial. In fact, several studies indicate that higher Treg infiltration is associated with a favorable prognosis in this tumor type [42, 43], possibly due to their role in controlling excessive inflammation and maintaining tissue homeostasis. Our data suggest that the Treg subsets expanded in CMAS KO tumors may be less immunosuppressive, as indicated by their less differentiated phenotype observed in the wanderlust trajectory analysis. This could explain why increased Treg numbers do not correlate with enhanced tumor growth in this context. Remarkably, KLRG1^+^CD103^+^ γδ T cells and CD69^+^ KLRG1^+^ NK cells, both indicative of a dysfunctional state, were present at higher percentages in sialylated tumors and correlated with higher PCI scores. Accordingly, mice bearing CT26‐MOCK tumors accumulated more ascites, which is enriched for IL‐10 and TGF‐β [40, 41]. These cytokines can promote the differentiation of γδ T cells into a tumor‐favorable state [44], supporting cancer progression. Together, this might provide a mechanistic link between ascites formation, a higher number of peritoneal tumors, and ongoing immune suppression.

Compared with subcutaneous CT26 tumors [28], PMs harbored elevated numbers of neutrophils. Given that all neutrophil clusters associated with a high PCI score were significantly diminished in CMAS KO tumors, this implies that, in this context, neutrophils may possess pro‐tumorigenic properties, thus hindering antitumor immunity. In KO tumors, neutrophils negatively correlated with antigen‐presenting monocytes and macrophages, as well as NK cells. Neutrophils are known to impair NK cell function in CRC [45], suggesting that they might promote tumor progression in this model as well. There is substantial evidence that the TME of patients with distant CRC metastases contains a specific population of tumor‐associated neutrophils (TANs), which support tumor growth, invasion, and angiogenesis [46, 47, 48, 49]. The skewing of neutrophils toward a tumor‐supportive phenotype is regulated by TGF‐β [50]. Since PM ascites contains high levels of TGF‐β [41], this suggests that more ascites could support pro‐tumorigenic neutrophils. Nevertheless, the role of neutrophils in antitumor immunity remains controversial, with ongoing debates on whether TANs represent a distinct population or are a subset of MDSCs.

Besides granulocytes, the first immune cells to encounter tumor cells are typically macrophages and DCs, attracted by signals from the tumor or surrounding stromal cells [51]. Cross‐presenting cDC1s and pDCs negatively correlated with the PCI score and were prominently present in Sia‐devoid tumors, suggesting that these subsets play a crucial role in immunosurveillance. cDC1 abundance inversely correlated with CD206^+^ macrophages, and the monocytes_2 and myeloid_2 clusters, all of which may exhibit pro‐tumorigenic properties. Nevertheless, the most abundant cell types in CT26 PMs were macrophages and monocytes, the main cell types found in the peritoneal cavity of mice and humans [52, 53]. Remarkably, two MHC‐II^+^ macrophage clusters (macrophage_1 and macrophage_3), indicating an antigen‐presenting phenotype, were significantly enriched in CMAS KO tumors and positively correlated with PD‐1^+^ tumor‐specific CD8^+^ T cells. This suggests that pro‐inflammatory macrophages may interact with T cells, potentially locally re‐activating them [54, 55, 56]. The CMAS KO‐enriched MHC‐II^+^ monocytes_1 cluster may represent inflammatory monocytes capable of presenting antigens, through a process called “cross‐dressing”, to boost CD8^+^ T cell expansion [57]. In CMAS KO tumors, CD206^+^Siglec‐E^+^ macrophages (macrophages_4) were significantly reduced and associated with higher PCI scores. Tumor‐derived Sias have been shown to engage Siglec‐7/9 (humans) and Siglec‐E (mice), promoting immunosuppressive macrophage polarization [58, 59]. Consistently, CMAS KO tumors exhibited more antitumorigenic tumor‐associated macrophages (TAMs) and fewer CD206^+^Siglec‐E^+^ TAMs, suggesting a shift toward a pro‐inflammatory phenotype. This aligns with Stanczak et al. [59], who reported that Siglec‐E deletion enhances antitumor immunity and slows tumor growth.

However, we cannot exclude that this mechanism is restricted solely to CT26 tumors. It is well established that different tumor types respond differently to desialylation. For instance, Sia removal in a PDAC cell line did not significantly affect tumor growth in vivo, except for a modest effect when combined with immunotherapy [25]. In contrast, desialylation on MC38 tumors resulted in increased tumor growth in vivo [30]. These findings indicate that the impact of tumor desialylation is not a universal mechanism and may depend on the specific tumor type, as well as the inherent levels of sialylation.

In conclusion, our results demonstrate that complete elimination of Sias results in a more immune‐permissive TME at the peritoneal metastatic site, characterized by increased infiltration of lymphoid cells and antigen‐presenting cells. This rewiring of the immune landscape following tumor desialylation was paired with smaller and fewer peritoneal tumors and thus a lower PCI score. In this context, we highlight the importance of Sias as an immune checkpoint, preventing effective antitumor immunity in a peritoneal cancer model. Hence, targeting sialylation represents a promising strategy to enhance immune‐mediated tumor control in CRC with peritoneal metastasis.

### 3.1 Study Limitations and Future Perspectives

Most in vivo studies rely on syngeneic subcutaneous grafts, yet the immune landscape in subcutaneous tissue is evidently different from other organs, including the colon. Orthotopic CRC models present a promising alternative; however, the invasive nature of the surgical procedure often induces inflammation, leading to a nonphysiological TME. Moreover, orthotopic CRC tumors metastasize to the liver, not the peritoneum. We modeled peritoneal metastasis by injecting tumor cells into the peritoneal cavity, enabling assessment of tumor growth and the immune landscape at a physiologically relevant site distinct from the frequently used subcutaneous location. The most important drawback is that our model does not represent actual metastatic colonization from the primary colorectal tumor.

Therefore, investigating Sia modulation in a spontaneous metastatic model with an inducible CMAS knockout would be of particular interest. Future studies are needed to address the role and functional activity of individual immune subsets identified in our analyses, like Tregs and the different macrophage and neutrophil subtypes. Additionally, our approach to genetically engineer a CMAS KO is not feasible for clinical application. Other strategies to target the Sia‐Siglec axis, including the use of Sia inhibitors or blocking antibodies, should be considered to evaluate the translatability into the clinic.

## 4 Materials and Methods

### 4.1 Cell Lines and Cell Culture

The murine colorectal cancer CT26 cells (obtained from ATCC; RRID: CVCL_7254) were cultured in RPMI‐1640 medium supplemented with 10% FCS, 1% glutamine, and 1% pen/strep. CT26 sialic acid knockout (KO) cells were generated as previously described [30] and kept under selection using 10 µg/mL of puromycin. Loss of sialic acids in KO cells was confirmed by staining with 2 µg/mL of biotinylated‐α2‐3‐Lectenz (Lectenz Bio), preincubated with 5 µg/mL of streptavidin‐AF488 (Life Technologies) in HBSS Mg^2+^/Ca^2+^ with 0.5% BSA. 7‐Aminoactinomycin D (7AAD; Thermo Fisher) was included as a live/dead marker and measured on the BD LSRFortessa flow cytometer. All experiments were performed with mycoplasma‐free cells.

### 4.2 In Vivo Peritoneal Tumor Model

Female BALB/c mice (6–8 weeks; Charles River) were housed in groups of six at the Amsterdam UMC animal facility. Experiments followed national and international guidelines (AVD11400202216545). To model peritoneal metastasis, 10^4^ tumor cells were injected intraperitoneally in RPMI with 50% Matrigel (Corning). Mice were monitored and weighed three times per week and sacrificed after 17 days or earlier if severe discomfort occurred (>15% weight loss). Peritoneal metastases were evaluated as described by Bastiaenen et al. [29] using a murine PCI (mPCI) scoring system [60]. The peritoneal cavity was divided into seven regions (right and left subphrenic, (sub)hepatic, subgastric, small bowel/mesentery, pelvic and back area); each scored 0–3 based on nodule size and number (0 = none, 1 = ≤2 mm, 2 = 2–5 mm or >5 nodules, 3 = ≥5 mm or >10 nodules). The mPCI is the sum of all regions (max. 21). Tumors were isolated and pooled per mouse for further experiments.

### 4.3 Tissue Processing and Immune Cell Profiling

Tumors were digested as previously described by van der Haar et al. [28]. Antibody staining with lymphoid and myeloid panels (Tables S1 and S2) was performed on up to 3×10^6^ cells per well in 96‐well V‐bottom plates using PBS with 1% BSA at 4°C. Antibody mixes were prepared in Brilliant Stain Buffer (BD Biosciences) diluted 1:1 with PBS/1% BSA. For both panels, cells were washed with PBS and stained with LIVE/DEAD Fixable Blue and Fc‐block (clone 2.4G2; produced in‐house) for 15–20 min at 4°C, followed by staining with the respective antibody mixes in the presence of True‐Stain Monocyte Blocker (BioLegend) for 30 min on ice. For tetramer staining, PE‐conjugated gp70 tetramer (H‐2L(d) SPSYVYHQF; NIH Tetramer Core Facility, Emory University) was added prior to surface staining and incubated for 15 min at 37°C. For intracellular staining, cells were fixed and permeabilized according to the manufacturer's instructions (Foxp3 Transcription Factor Staining Buffer Set; eBioscience) and incubated with anti‐FoxP3‐PE‐CF594, anti‐CTLA‐4‐APC, and anti‐TCF‐1‐AF488 antibodies. For the myeloid panel, cells were fixed with 1% PFA for 20 min at 4°C. Data were acquired on a Cytek Aurora 5L spectral flow cytometer.

### 4.4 Flow Cytometry Data Analysis

Autofluorescence removal and data unmixing were performed using the SpectroFlo‐5L software. Dimensionality reduction and clustering were done in OMIQ, with quality control via the PeacoQC algorithm. For both the lymphoid and myeloid panels, CD45^+^ live single cells were manually gated. After conventional marker analysis, down‐sampling selected the maximal number of cells per tissue. Fluorescence minus one (FMO) samples confirmed marker expression and validated the gating. Dimensionality reduction was performed using t‐SNE‐CUDA, optSNE, or UMAP, followed by clustering with PARC or FLOWSOM. Clusters were merged and annotated based on marker expression. Wanderlust trajectory analysis was applied for specific lymphoid populations [61].

### 4.5 Bioinformatics Analysis

To compare sialylation‐related gene expression between primary CRC, liver, and peritoneal metastases, we made use of the RNAseq dataset (GSE190609) from Laoukili et al. [6]. Log2‐transformed RPKM values were compared using the R2: Genomics Analysis and Visualization Platform (http://r2.amc.nl) and Graphpad Prism 9.5.1 (pairwise comparisons with a mixed linear effects model). For the correlation heatmaps, Spearman correlation coefficients for various immune populations were calculated in R Studio using the corr.test function from the psych package, and the results were visualized with the corrplot package.

### 4.6 Statistical Analysis

Statistical significance was tested using GraphPad Prism 9 by performing unpaired nonparametric *t*‐tests and two‐way ANOVA with multiple comparison tests (*p < 0.05, **p < 0.01; ***p < 0.001; ****p < 0.0001; ns: not significant).

## Author Contributions

**I.v.d.H.A**.: Conceptualization, investigation, methodology, formal analysis, writing – original draft, writing – review and editing, visualization. **K.L**.: investigation, methodology, writing – review and editing. **V.L**.: investigation, methodology, writing – review and editing. **E.N**.: investigation; writing – review and editing. **E.R**.: formal analysis, writing – review and editing. **J.J.G.V**.: investigation, formal analysis, writing – review and editing. **Y.v.K**.: investigation, writing – review and editing. **J.G**.: investigation, writing – review and editing. **S.v.V**.: conceptualization, investigation, writing – original draft, writing – review and editing, funding acquisition, supervision.

## Funding

This work was financially supported by the Dutch Cancer Society to I.v.d.H.A. and V.L. (KWF, project grant 12420), to S.J.v.V (KWF project grant 16816), to K.L. (KWF project grant 13435/2021‐1), and to J.G. (KWF YIG 13915). J.G. was further supported by NWO ZonMw Vidi Grant 09150172210058, Top Institute for Knowledge and Innovation grant ImPACT, donation by Mr. H.J.M. Roels through the Oncode Institute.

## Ethics Statement

All animal experiments were approved by the Animal Experiments Committee of the VU University (DEC201512; CCD permit AVD11400202216545; protocol MCB22‐16545‐3‐06) and performed in accordance with national and international guidelines and regulations.

## Conflicts of Interest

The authors declare no conflicts of interest.

## Supporting information




**Supporting File**: eji70204‐sup‐0001‐SuppMat.pdf.

## Data Availability

All data that support the findings of this study are available from the corresponding author upon reasonable request.

## References

[1] E. Pretzsch , F. Bösch , J. Neumann , et al., “Mechanisms of Metastasis in Colorectal Cancer and Metastatic Organotropism: Hematogenous versus Peritoneal Spread,” Journal of Oncology 2019 (2019): 7407190, 10.1155/2019/7407190. 31641356 PMC6770301

[2] J. Franko , Q. Shi , J. P. Meyers , et al., “Prognosis of Patients With Peritoneal Metastatic Colorectal Cancer Given Systemic Therapy: An Analysis of Individual Patient Data From Prospective Randomised Trials From the Analysis and Research in Cancers of the Digestive System (ARCAD) Database,” The Lancet Oncology 17, no. 12 (2016): 1709–1719, 10.1016/S1470-2045(16)30500-9. 27743922

[3] Y. R. van Gestel , I. Thomassen , V. E. Lemmens , et al., “Metachronous Peritoneal Carcinomatosis After Curative Treatment of Colorectal Cancer,” European Journal of Surgical Oncology 40, no. 8 (2014): 963–969. 24183168 10.1016/j.ejso.2013.10.001

[4] K. J. Lenos , S. Bach , and L. Ferreira Moreno , “Molecular Characterization of Colorectal Cancer Related Peritoneal Metastatic Disease,” Nature Communications 13, no. 1 (2022): 4443, 10.1038/s41467-022-32198-z. PMC9352687 35927254

[5] J. Guinney , R. Dienstmann , X. Wang , et al., “The Consensus Molecular Subtypes of Colorectal Cancer,” Nature Medicine 21, no. 11 (2015): 1350–1356, 10.1038/nm.3967. PMC4636487 26457759

[6] J. Laoukili , A. Constantinides , E. C. E. Wassenaar , et al., “Peritoneal Metastases From Colorectal Cancer Belong to Consensus Molecular Subtype 4 and Are Sensitised to Oxaliplatin by Inhibiting Reducing Capacity,” British Journal of Cancer 126, no. 12 (2022): 1824–1833, 10.1038/s41416-022-01742-5. 35194192 PMC9174226

[7] J. Kleber , J. Yang Zhou , and F. Weber , “Immune Profile of Patients With Peritoneal Carcinomatosis Selected for CRS‐HIPEC Therapy,” Cancer Immunology, Immunotherapy 72, no. 11 (2023): 3867–3873, 10.1007/s00262-023-03515-2. 37580610 PMC10576707

[8] P. Sundström , S. Hogg , M. Quiding Järbrink , and E. Bexe Lindskog , “Immune Cell Infiltrates in Peritoneal Metastases From Colorectal Cancer,” Frontiers in Immunology 15 (2024): 1347900, 10.3389/fimmu.2024.1347900. 38384469 PMC10879551

[9] K. Boelaars , E. Rodriguez , Z. R. Huinen , et al., “Pancreatic Cancer‐Associated Fibroblasts Modulate Macrophage Differentiation via Sialic Acid‐Siglec Interactions,” Communications Biology 7, no. 1 (2024): 430, 10.1038/s42003-024-06087-8. 38594506 PMC11003967

[10] P. Chiodelli , S. Rezzola , and C. Urbinati , “Contribution of Vascular Endothelial Growth Factor Receptor‐2 Sialylation to the Process of Angiogenesis,” Oncogene 36, no. 47 (2017): 6531–6541, 10.1038/onc.2017.243. 28783175

[11] P. R. Crocker , J. C. Paulson , and A. Varki , “Siglecs and Their Roles in the Immune System,” Nature Reviews Immunology 7, no. 4 (2007): 255–266, 10.1038/nri2056. 17380156

[12] J. E. Hudak , S. M. Canham , and C. R. Bertozzi , “Glycocalyx Engineering Reveals a Siglec‐Based Mechanism for NK Cell Immunoevasion,” Nature Chemical Biology 10, no. 1 (2014): 69–75, 10.1038/nchembio.1388. 24292068 PMC3893890

[13] C. Jandus , K. F. Boligan , O. Chijioke , et al., “Interactions Between Siglec‐7/9 Receptors and Ligands Influence NK Cell‐Dependent Tumor Immunosurveillance,” Journal of Clinical Investigation 124, no. 4 (2014): 1810–1820, 10.1172/JCI65899. 24569453 PMC3973073

[14] J. Daly , S. Sarkar , A. Natoni , et al., “Targeting Hypersialylation in Multiple Myeloma Represents a Novel Approach to Enhance NK Cell‐Mediated Tumor Responses,” Blood Advances 6, no. 11 (2022): 3352–3366, 10.1182/bloodadvances.2021006805. 35294519 PMC9198929

[15] J. Wang , J. Sun , L. N. Liu , et al., “Siglec‐15 as an Immune Suppressor and Potential Target for Normalization Cancer Immunotherapy,” Nature Medicine 25, no. 4 (2019): 656–666, 10.1038/s41591-019-0374-x. PMC7175920 30833750

[16] J. Wang , M. Manni , A. Bärenwaldt , et al., “Siglec Receptors Modulate Dendritic Cell Activation and Antigen Presentation to T Cells in Cancer,” Frontiers in Cell and Developmental Biology 10 (2022): 828916, 10.3389/fcell.2022.828916. 35309936 PMC8927547

[17] Y. Ding , Z. Guo , Y. Liu , et al., “The Lectin Siglec‐G Inhibits Dendritic Cell Cross‐Presentation by Impairing MHC Class I‐Peptide Complex Formation,” Nature Immunology 17, no. 10 (2016): 1167–1175, 10.1038/ni.3535. 27548433

[18] M. Perdicchio , J. M. Ilarregui , M. I. Verstege , et al., “Sialic Acid‐Modified Antigens Impose Tolerance via Inhibition of T‐Cell Proliferation and De Novo Induction of Regulatory T Cells,” Proceedings of National Academy of Sciences 113, no. 12 (2016): 3329–3334, 10.1073/pnas.1507706113. PMC4812702 26941238

[19] A. A. Barkal , R. E. Brewer , M. Markovic , et al., “CD24 Signalling Through Macrophage Siglec‐10 Is a Target for Cancer Immunotherapy,” Nature 572, no. 7769 (2019): 392–396, 10.1038/s41586-019-1456-0. 31367043 PMC6697206

[20] R. Beatson , V. Tajadura‐Ortega , D. Achkova , et al., “The Mucin MUC1 Modulates the Tumor Immunological Microenvironment Through Engagement of the Lectin Siglec‐9,” Nature Immunology 17, no. 11 (2016): 1273–1281, 10.1038/ni.3552. 27595232 PMC5257269

[21] H. Läubli , O. M. Pearce , F. Schwarz , et al., “Engagement of Myelomonocytic Siglecs by Tumor‐Associated Ligands Modulates the Innate Immune Response to Cancer,” Proceedings of National Academy of Sciences 111, no. 39 (2014): 14211–14216, 10.1073/pnas.1409580111. PMC4191788 25225409

[22] M. Perdicchio , L. A. Cornelissen , I. Streng‐Ouwehand , et al., “Tumor Sialylation Impedes T Cell Mediated Anti‐Tumor Responses While Promoting Tumor Associated‐Regulatory T Cells,” Oncotarget 7, no. 8 (2016): 8771–8782, 10.18632/oncotarget.6822. 26741508 PMC4891003

[23] C. Büll , T. J. Boltje , N. Balneger , et al., “Sialic Acid Blockade Suppresses Tumor Growth by Enhancing T‐Cell‐Mediated Tumor Immunity,” Cancer Research 78, no. 13 (2018): 3574–3588. 29703719 10.1158/0008-5472.CAN-17-3376

[24] M. A. Gray , M. A. Stanczak , N. R. Mantuano , et al., “Targeted Glycan Degradation Potentiates the Anticancer Immune Response in Vivo,” Nature Chemical Biology 16, no. 12 (2020): 1376–1384, 10.1038/s41589-020-0622-x. 32807964 PMC7727925

[25] K. Boelaars , L. Goossens‐Kruijssen , D. Wang , et al., “Unraveling the Impact of Sialic Acids on the Immune Landscape and Immunotherapy Efficacy in Pancreatic Cancer,” Journal for ImmunoTherapy of Cancer 11, no. 11 (2023): e007805, 10.1136/jitc-2023-007805. 37940346 PMC10632901

[26] H. Läubli and L. Borsig , “Altered Cell Adhesion and Glycosylation Promote Cancer Immune Suppression and Metastasis,” Frontiers in Immunology 10 (2019): 2120, 10.3389/fimmu.2019.02120. 31552050 PMC6743365

[27] C. Dobie and D. Skropeta , “Insights Into the Role of Sialylation in Cancer Progression and Metastasis,” British Journal of Cancer 124, no. 1 (2021): 76–90, 10.1038/s41416-020-01126-7. 33144696 PMC7782833

[28] H. van der , I. Àvila , T. Zhang , et al., “Tumor Desialylation Surpasses Anti‐PD‐L1 Checkpoint Therapy in Restoring Anti‐Tumor Immunity in a Murine Model for Colorectal Cancer,” International Journal of Cancer 157, no. 9 (2025): 1948–1962, 10.1002/ijc.70031. 40622037 PMC12407041

[29] V. P. Bastiaenen , C. E. L. Klaver , M. C. S. van der Heijden , et al., “A Mouse Model for Peritoneal Metastases of Colorectal Origin Recapitulates Patient Heterogeneity,” Laboratory Investigation 100, no. 11 (2020): 1465–1474, 10.1038/s41374-020-0448-x. 32504005

[30] L. A. M. Cornelissen , A. Blanas , and J. C. van der Horst , “Disruption of Sialic Acid Metabolism Drives Tumor Growth by Augmenting CD8(+) T Cell Apoptosis,” International Journal of Cancer 144, no. 9 (2019): 2290–2302, 10.1002/ijc.32084. 30578646 PMC6519079

[31] S. L. Sangisetty and T. J. Miner , “Malignant Ascites: A Review of Prognostic Factors, Pathophysiology and Therapeutic Measures,” World Journal of Gastrointestinal Surgery 4, no. 4 (2012): 87–95, 10.4240/wjgs.v4.i4.87. 22590662 PMC3351493

[32] J. M. Wang , Y. Q. Cheng , L. Shi , et al., “KLRG1 Negatively Regulates Natural Killer Cell Functions Through the Akt Pathway in Individuals With Chronic hepatitis C Virus Infection,” Journal of Virology 87, no. 21 (2013): 11626–11636, 10.1128/JVI.01515-13. 23966413 PMC3807337

[33] Y. Mei , X. Wang , J. Zhang , et al., “Siglec‐9 Acts as an Immune‐Checkpoint Molecule on Macrophages in Glioblastoma, Restricting T‐Cell Priming and Immunotherapy Response,” Nature Cancer 4, no. 9 (2023): 1273–1291, 10.1038/s43018-023-00598-9. 37460871

[34] J. G. Rodrigues , M. Balmaña , J. A. Macedo , et al., “Glycosylation in Cancer: Selected Roles in Tumour Progression, Immune Modulation and Metastasis,” Cellular Immunology 333 (2018): 46–57, 10.1016/j.cellimm.2018.03.007. 29576316

[35] E. RodrÍguez , S. T. T. Schetters , and Y. van Kooyk , “The Tumour Glyco‐Code as a Novel Immune Checkpoint for Immunotherapy,” Nature Reviews Immunology 18, no. 3 (2018): 204–211, 10.1038/nri.2018.3. 29398707

[36] S. S. Pinho and C. A. Reis , “Glycosylation in Cancer: Mechanisms and Clinical Implications,” Nature Reviews Cancer 15, no. 9 (2015): 540–555, 10.1038/nrc3982. 26289314

[37] S. T. Teoh , M. P. Ogrodzinski , C. Ross , K. W. Hunter , and S. Y. Lunt , “Sialic Acid Metabolism: A Key Player in Breast Cancer Metastasis Revealed by Metabolomics,”Frontiers in Oncology 8 (2018): 174, 10.3389/fonc.2018.00174. 29892572 PMC5985449

[38] H. Yamanami , K. Shiozaki , T. Wada , et al., “Down‐Regulation of Sialidase NEU4 May Contribute to Invasive Properties of Human Colon Cancers,” Cancer Science 98, no. 3 (2007): 299–307, 10.1111/j.1349-7006.2007.00403.x. 17270019 PMC11159927

[39] F. Dall'Olio , M. Pucci , and N. Malagolini , “The Cancer‐Associated Antigens Sialyl Lewis(a/x) and Sd(a): Two Opposite Faces of Terminal Glycosylation,” Cancers (Basel) 13, no. 21 (2021): 5273, 10.3390/cancers13215273. 34771437 PMC8582462

[40] J. Saris , S. Bootsma , J. Verhoeff , et al., “T‐cell Responses in Colorectal Peritoneal Metastases Are Recapitulated in a Humanized Immune System Mouse Model,” Frontiers in Immunology 15 (2024): 1415457, 10.3389/fimmu.2024.1415457. 39044825 PMC11263213

[41] E. Küçükköse , B. A. Heesters , J. Villaudy , et al., “Modeling Resistance of Colorectal Peritoneal Metastases to Immune Checkpoint Blockade in Humanized Mice,” Journal for ImmunoTherapy of Cancer 10, no. 12 (2022): e005345. 36543378 10.1136/jitc-2022-005345 PMC9772695

[42] T. Hanke , N. Melling , R. Simon , et al., “High Intratumoral FOXP3^+^ T Regulatory Cell (Tregs) Density Is an Independent Good Prognosticator in Nodal Negative Colorectal Cancer,” International Journal of Clinical and Experimental Pathology 8, no. 7 (2015): 8227–8235. 26339391 PMC4555719

[43] B. Shang , Y. Liu , J. S‐j , and Y. Liu , “Prognostic Value of Tumor‐infiltrating FoxP3+ Regulatory T Cells in Cancers: A Systematic Review and Meta‐analysis,” Scientific Reports 5, no. 1 (2015): 15179, 10.1038/srep15179. 26462617 PMC4604472

[44] Z. Gao , Y. Bai , A. Lin , et al., “Gamma Delta T‐Cell‐Based Immune Checkpoint Therapy: Attractive Candidate for Antitumor Treatment,” Molecular Cancer 22, no. 1 (2023): 31, 10.1186/s12943-023-01722-0. 36793048 PMC9930367

[45] Y. Zhang , Z. Wang , Y. Lu , et al., “Region‐Specific CD16(+) Neutrophils Promote Colorectal Cancer Progression by Inhibiting Natural Killer Cells,” Advances in Science (Weinh) 11, no. 29 (2024): e2403414, 10.1002/advs.202403414. PMC11304263 38790136

[46] R. Mizuno , K. Kawada , Y. Itatani , R. Ogawa , Y. Kiyasu , and Y. Sakai , “The Role of Tumor‐Associated Neutrophils in Colorectal Cancer,” International Journal of Molecular Sciences 20, no. 3 (2019): 529, 10.3390/ijms20030529. 30691207 PMC6386937

[47] T. M. Bui , L. K. Yalom , E. Ning , et al., “Tissue‐specific Reprogramming Leads to Angiogenic Neutrophil Specialization and Tumor Vascularization in Colorectal Cancer,” The Journal of Clinical Investigation 134, no. 7 (2024): e174545, 10.1172/JCI174545. 38329810 PMC10977994

[48] X. Huang , E. Nepovimova , V. Adam , et al., “Neutrophils in Cancer Immunotherapy: Friends or Foes?,” Molecular Cancer 23, no. 1 (2024): 107, 10.1186/s12943-024-02004-z. 38760815 PMC11102125

[49] S. Tian , Y. Chu , J. Hu , et al., “Tumour‐Associated Neutrophils Secrete AGR2 to Promote Colorectal Cancer Metastasis via Its Receptor CD98hc‐xCT,” Gut 71, no. 12 (2022): 2489–2501, 10.1136/gutjnl-2021-325137. 35086885

[50] Z. G. Fridlender , J. Sun , S. Kim , et al., “Polarization of Tumor‐Associated Neutrophil Phenotype by TGF‐beta: “N1” versus “N2” TAN,” Cancer Cell 16, no. 3 (2009): 183–194, 10.1016/j.ccr.2009.06.017. 19732719 PMC2754404

[51] C. Li , X. Yu , X. Han , et al., “Innate Immune Cells in Tumor Microenvironment: A New Frontier in Cancer Immunotherapy,” Iscience 27, no. 9 (2024): 110750, 10.1016/j.isci.2024.110750. 39280627 PMC11399700

[52] X.‐Z. Huang , M.‐J. Pang , J.‐Y. Li , et al., “Single‐Cell Sequencing of Ascites Fluid Illustrates Heterogeneity and Therapy‐Induced Evolution During Gastric Cancer Peritoneal Metastasis,” Nature Communications 14, no. 1 (2023): 822, 10.1038/s41467-023-36310-9. PMC9929081 36788228

[53] J. Han , A. Gallerand , E. C. Erlich , et al., “Human Serous Cavity Macrophages and Dendritic Cells Possess Counterparts in the Mouse With a Distinct Distribution Between Species,” Nature Immunology 25, no. 1 (2024): 155–165, 10.1038/s41590-023-01688-7. 38102487 PMC10990619

[54] G. J. L. Macrophages , “Their Untold Story in T Cell Activation and Function,” International Review of Cell and Molecular Biology 342 (2019): 73–93. 30635094 10.1016/bs.ircmb.2018.07.001

[55] J. Waibl Polania , A. Hoyt‐Miggelbrink , and W. H. Tomaszewski , “Antigen Presentation by Tumor‐Associated Macrophages Drives T Cells From a Progenitor Exhaustion State to Terminal Exhaustion,” Immunity 58, no. 1 (2025): 232–246, 10.1016/j.immuni.2024.11.026. 39724910

[56] P. Meiser , M. A. Knolle , A. Hirschberger , et al., “A Distinct Stimulatory cDC1 Subpopulation Amplifies CD8(+) T Cell Responses in Tumors for Protective Anti‐Cancer Immunity,” Cancer Cell 41, no. 8 (2023): 1498–1515.e10, 10.1016/j.ccell.2023.06.008. 37451271

[57] A. Elewaut , G. Estivill , F. Bayerl , et al., “Cancer Cells Impair Monocyte‐Mediated T Cell Stimulation to Evade Immunity,” Nature 637, no. 8046 (2025): 716–725, 10.1038/s41586-024-08257-4. 39604727 PMC7617236

[58] E. Rodriguez , K. Boelaars , and K. Brown , “Sialic Acids in Pancreatic Cancer Cells Drive Tumour‐Associated Macrophage Differentiation via the Siglec Receptors Siglec‐7 and Siglec‐9,” Nature Communications 12, no. 1 (2021): 1270, 10.1038/s41467-021-21550-4. PMC7904912 33627655

[59] M. A. Stanczak , N. Rodrigues Mantuano , and N. Kirchhammer , “Targeting Cancer Glycosylation Repolarizes Tumor‐Associated Macrophages Allowing Effective Immune Checkpoint Blockade,” Science Translational Medicine 14, no. 669 (2022): eabj1270, 10.1126/scitranslmed.abj1270. 36322632 PMC9812757

[60] P. Jacquet , P. H. Sugarbaker , and P. H. Sugarbaker , Clinical Research Methodologies in Diagnosis and Staging of Patients With Peritoneal Carcinomatosis, Peritoneal Carcinomatosis: Principles of Management (Springer US, 1996), 359–374. 10.1007/978-1-4613-1247-5_23 8849962

[61] S. C. Bendall , K. L. Davis , and A. D. Amir el , “Single‐cell Trajectory Detection Uncovers Progression and Regulatory Coordination in human B Cell Development,” Cell 157, no. 3 (2014): 714–725, 10.1016/j.cell.2014.04.005. 24766814 PMC4045247

