# Supplementary material for: Sialylation in Colorectal Cancer Rewires Antitumor Immunity at the Peritoneal Metastatic Site
Source: Eur J Immunol. 2026 May 19;56:e70204. doi: 10.1002/eji.70204 (PMC13185675; doi:10.1002/eji.70204)
Supplement: Supplementary file 1 — Supporting File: eji70204‐sup‐0001‐SuppMat.pdf. [file EJI-56-e70204-s001.pdf]

Supporting information

Sialylation in Colorectal Cancer Rewires Anti-Tumor Immunity at the Peritoneal Metastatic Site

Irene van der Haar Àvila[1,2,3], Kristiaan Lenos[4,5], Victor Lorrain[1,2,3], Eleonora Nardini[1,2,3], Ernesto Rodríguez[1,2,3], Juan J. García-Vallejo[1,2,3], Yvette van Kooyk[1,2,3], Joep Grootjans[2,4,5,6,7], Sandra J. van Vliet[1,2,3]

[1]Amsterdam UMC location Vrije Universiteit Amsterdam, Department of Molecular Cell Biology and Immunology, de Boelelaan 1117, Amsterdam, the Netherlands

[2]Cancer Center Amsterdam, Cancer Biology and Immunology, Amsterdam, the Netherlands

[3]Amsterdam institute for Immunology and Infectious Diseases, Cancer Immunology, Amsterdam, the Netherlands

[4]Amsterdam UMC location Vrije Universiteit Amsterdam, Center for Experimental and Molecular Medicine, Laboratory for Experimental Oncology and Radiobiology, van der Boechorststraat 6A, Amsterdam, the Netherlands

[5]Oncode Institute, Amsterdam, the Netherlands

[6]Department of Gastroenterology and Hepatology, Amsterdam UMC, location Vrije Universiteit Amsterdam, de Boelelaan 1117, Amsterdam, the Netherlands

[7]Amsterdam Gastroenterology Endocrinology Metabolism, Amsterdam, the Netherlands

Table S1. Detailed information of mouse lymphoid antibodies.

Marker	Fluorochrome	Clone	Company	Cat#	Titration
CD45	cFluor V547	30F11	Cytek Biosciences	RC-00426	1:50
CD3	BUV805	17A2	BD Biosciences	741982	1:25
CD4	BUV395	RM4-5	BD Biosciences	740208	1:200
CD8b	BUV563	H35-17.2	BD Biosciences	741342	1:200
TCRγδ	Super Bright 780	GL3	Cytek Biosciences	SKU 78-5711-82	1:400
FoxP3	PE-CF594	3G3	BD Biosciences	567456	1:25
CD103	PECy5	2E7	Biolegend	121448	1:200
CD44	cFluor BYG710	IM7	Cytek Biosciences	RC-00514	1:800
PD-1 (CD279)	BV421	29F.1A12	Biolegend	135217	1:25
CD25	PE-Cy7	PC61.5	Cytek Biosciences	60-0251-U100	1:400
CD62L	cFluor R720	MEL-14	Cytek Biosciences	RC-00515	1:800
TIM-3 (CD366)	BV480	5D12/TIM-3	BD Biosciences	747618	1:200
CD69	BUV737	H1.2F3	BD Biosciences	612793	1:200
CD39	Super Bright 702	24DMS1	Cytek Biosciences	SKU 67-0391-82	1:400
CD19	BUV615	1D3	BD Biosciences	751213	1:100
CTLA-4 (CD152)	APC	UC10-4B9	Cytek Biosciences	20-1522-U100	1:800
KLRG-1	cFluor R780	2F1	Cytek Biosciences	RC-00516	1:50
TCF-1	AF488	S33-966	BD Biosciences	567018	1:100
CX3CR1	BV605	SA011F11	Biolegend	149027	1:1000
NKp46 (CD335)	BV750	29A1.4	BD Biosciences	746875	1:50
gp70 tetramer	PE		NIH		1:100
LIVE/DEAD™ Fixable Blue Dead Cell Stain Kit			ThermoFisher	L34961	1:1000

Table S2. Detailed information of mouse myeloid antibodies.

Marker	Fluorochrome	Clone	Company	Cat#	Titration
CD45	cFluor V547	30-F11	Cytek Biosciences	RC-00426	1:50
CD11c	cFluor V450	N418	Cytek Biosciences	RC-00509	1:50
Siglec-F (CD170)	BV480	E50-2440	BD Biosciences	746668	1:400
CD64	PE-Dazzle594	X54-5/7.1	Biolegend	139319	1:100
Ly6G	BUV563	1A8	BD Biosciences	612921	1:50
F4/80	Super Bright 780	BM8	Cytek Biosciences	78-4801-82	1:800
XCR1	BV650	ZET	Biolegend	148220	1:25
MHC-II (I-A/I-E)	BUV395	2G9	BD Biosciences	743876	1:200
CD11b	cFluor R840	M1/70	Cytek Biosciences	RC-00510	1:800
Ly6C	cFluor B548	HK1.4	Cytek Biosciences	RC-00511	1:200
CD206	PE	MR6F3	ThermoFisher	12-2061-82	1:800
CD103	PECy5	2E7	Biolegend	121448	1:200
PD-L1 (CD274)	cFluor BYG710	10F.9G2	Cytek Biosciences	RC-00447	1:800
NKp46 (CD335)	BV510	29A1.4	Biolegend	137623	1:50
CD3	BV510	17A2	Biolegend	100233	1:50
CD19	BV510	6D5	Biolegend	115545	1:400
SIRPα (CD172a)	BV711	P84	BD Biosciences	740766	1:200
CCR2 (CD192)	BV421	SA203G11	Biolegend	150605	1:100
Siglec-E	PE-Cy7	M1304A01	Biolegend	677107	1:25
Siglec-H	BUV737	440c	BD Biosciences	748293	1:200
CD88	PerCP-Cy5.5	20/70	Biolegend	135812	1:200
CD26	VioBright FITC	H194-112	ThermoFisher	130-106-398	1:200
Siglec-15	AF647	MK4.5	Biolegend (conjugated in house)	164602	1:200
LIVE/DEAD™ Fixable Blue Dead Cell Stain Kit			ThermoFisher	L34961	1:1000

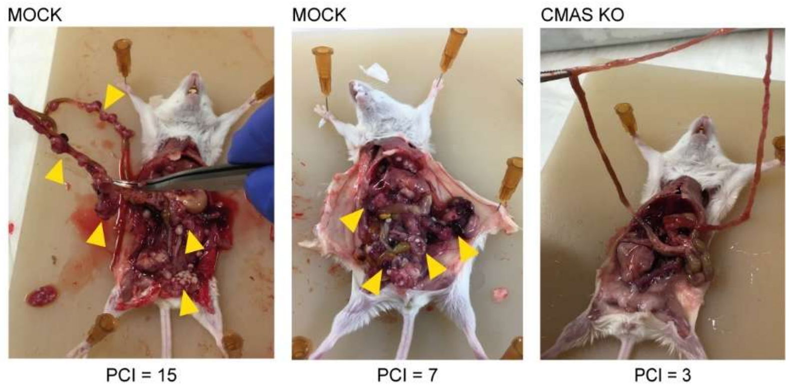

Figure S1. Mice with Sia-devoid CT26 tumors have less tumors in the peritoneum. Example images at day 17 post tumor inoculation with the PCI score of tumor outgrowth in MOCK and CMAS KO mice (*n*=20). Arrow heads indicate where the tumors are located in the peritoneum.

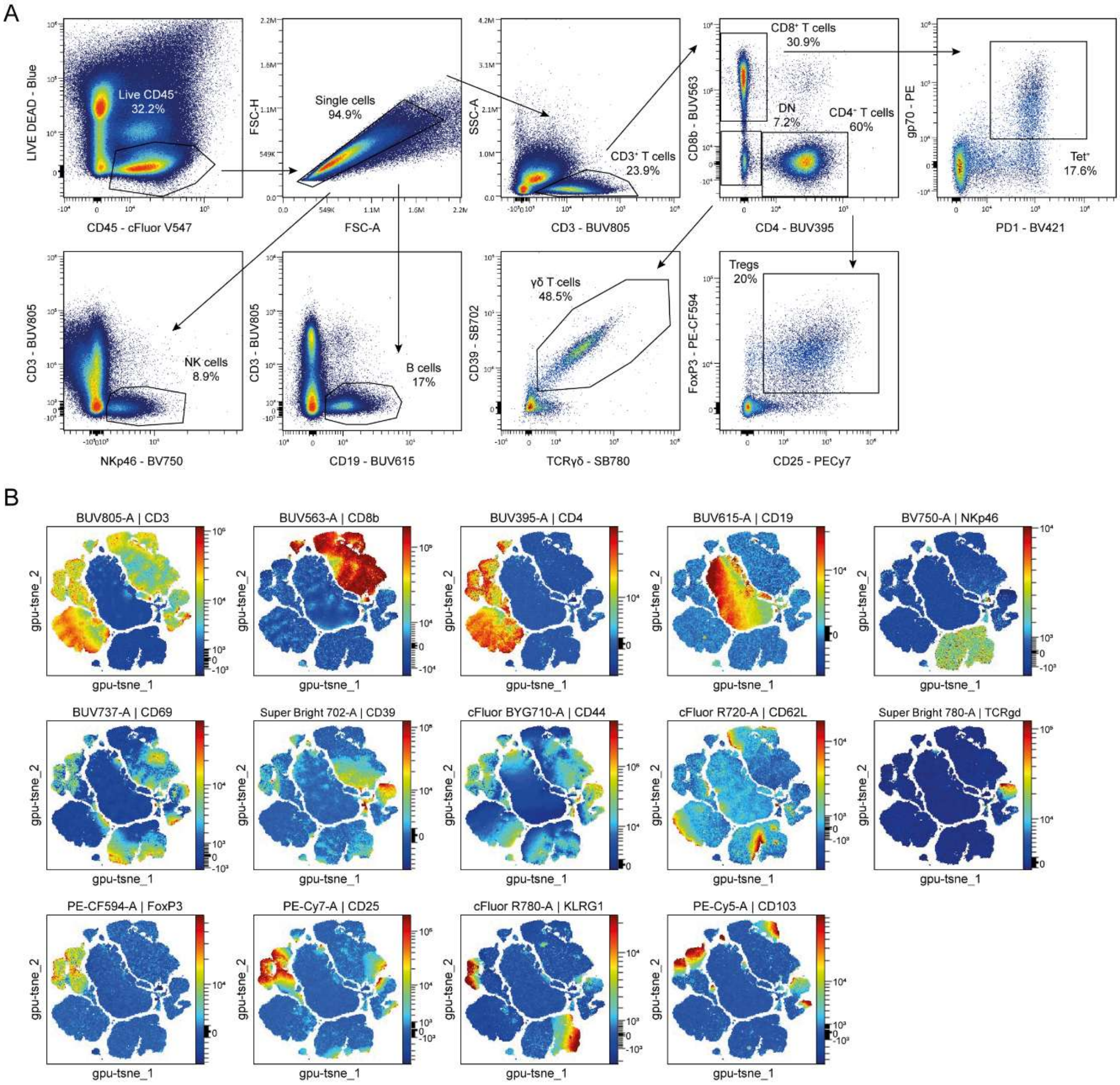

Figure S2. Expression of lymphoid markers in CT26 tumors. (**A**) Manual gating strategy for lymphoid populations in the TME, based on CD45⁺ alive cells and excluding doublets and myeloid cells by gating on either CD3⁺ T cells, NK cells or B cells. The marker expression and gating strategy was confirmed with FMO controls. (**B**) tSNE plots showing the expression of different lymphoid markers used for unsupervised clustering. Cells were gated on CD45⁺ alive immune cells after excluding the myeloid population.

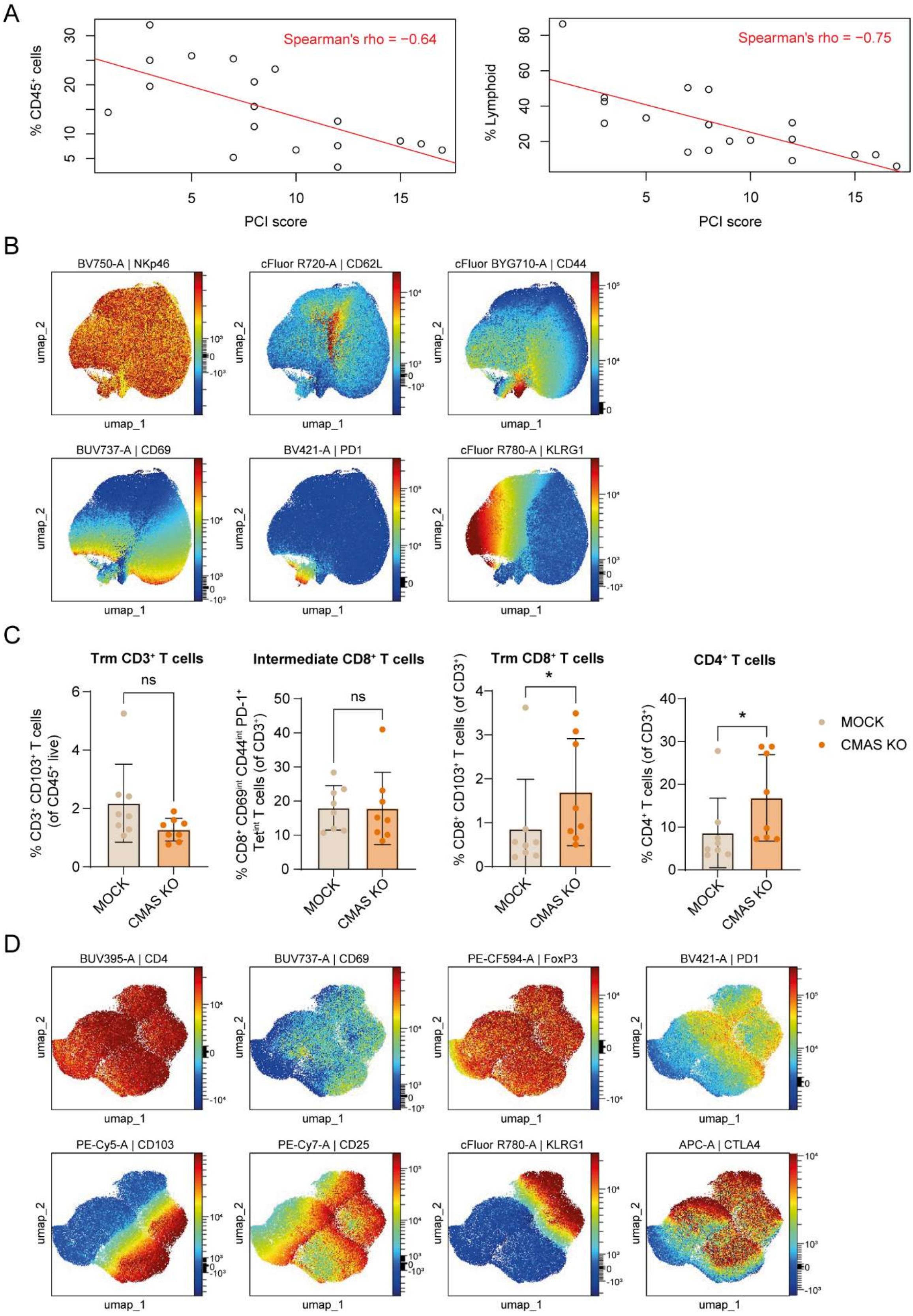
A
Spearman's rho = −0.64
% CD45⁺ cells
PCI score
Spearman's rho = −0.75
% Lymphoid
PCI score
B
BV750-A | NKp46
cFluor R720-A | CD62L
cFluor BYG710-A | CD44
BUV737-A | CD69
BV421-A | PD1
cFluor R780-A | KLRG1
umap_1
umap_2
C
Trm CD3⁺ T cells
% CD3⁺ CD103⁺ T cells (of CD45⁺ live)
ns
Intermediate CD8⁺ T cells
% CD8⁺ CD69int CD44int PD-1⁺ Tetint T cells (of CD3⁺)
ns
Trm CD8⁺ T cells
% CD8⁺ CD103⁺ T cells (of CD3⁺)
*
CD4⁺ T cells
% CD4⁺ T cells (of CD3⁺)
*
MOCK
CMAS KO
D
BUV395-A | CD4
BUV737-A | CD69
PE-CF594-A | FoxP3
BV421-A | PD1
PE-Cy5-A | CD103
PE-Cy7-A | CD25
cFluor R780-A | KLRG1
APC-A | CTLA4
umap_1
umap_2

Figure S3. The PCI score negatively correlates with the infiltration of $CD45^+$ immune and lymphoid cells. (**A**) Spearman correlation plots between the PCI score and the percentage of $CD45^+$ live cells or the percentage of lymphoid ($CD3^+$, $CD19^+$, $NKp46^+$) cells, respectively. (**B**) UMAP plots showing the expression of different NK cell markers found in the NK clusters, which were used for the wanderlust trajectory analysis. (**C**) Percentage of distinct $CD3^+$, $CD8^+$ and $CD4^+$ T cell clusters found in the TME of CT26-MOCK and CMAS KO tumors. Each dot represents one individual mouse. Data is shown as mean ± SD; unpaired non-parametric *t*-test (ns, not significant; * p≤0.05; ** p≤0.01). (**D**) UMAP plots showing the expression of Tregs markers found in the different subsets, which were used for the wanderlust trajectory analysis.

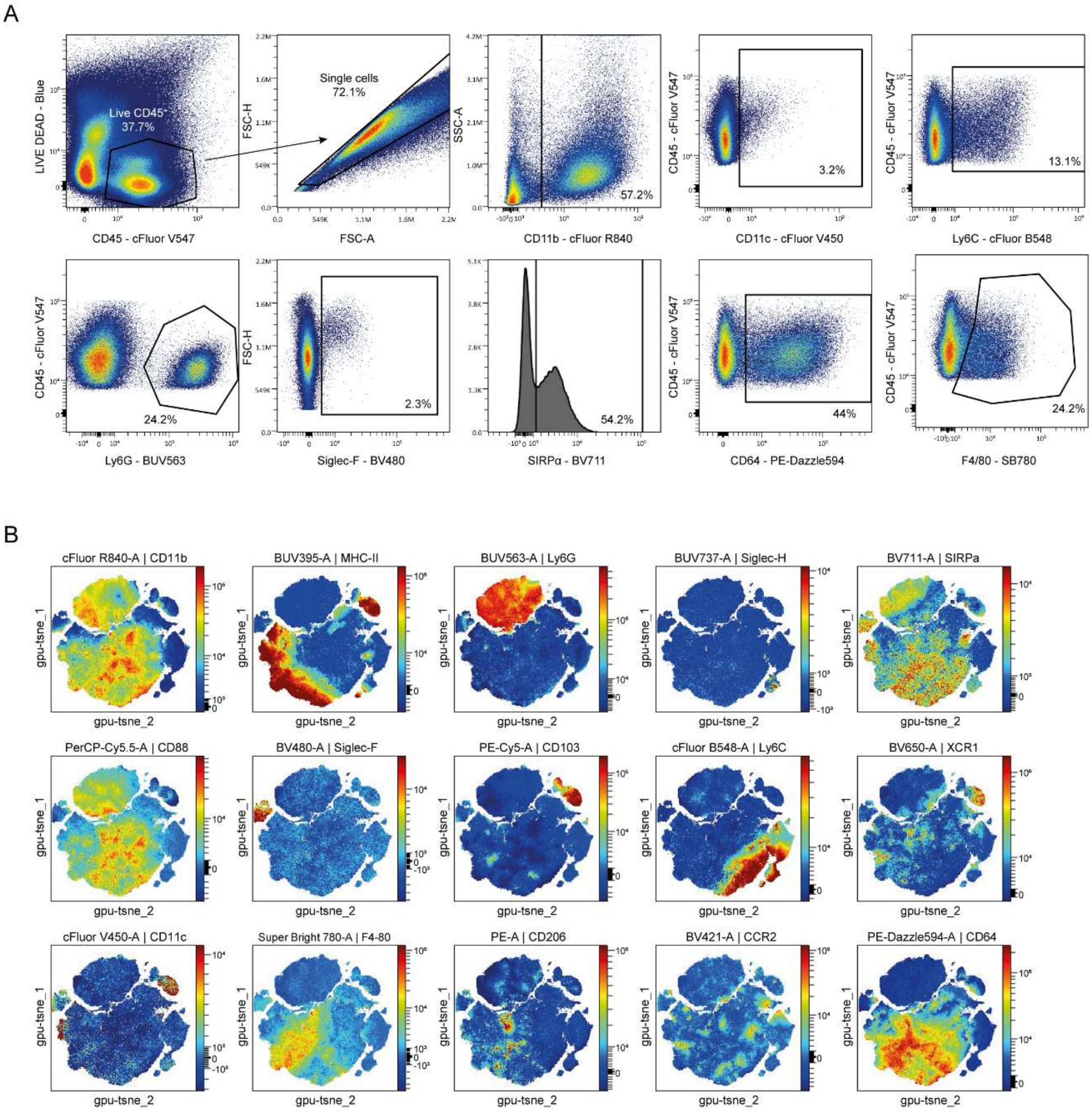

Figure S4. Expression of myeloid lineage markers in CT26 tumors. (**A**) Manual gating of myeloid lineage markers based on CD45$^+$ alive cells and used to exclude doublets and the lymphoid populations. The marker expression was confirmed with FMO controls. (**B**) tSNE plots showing the expression of the different myeloid markers used for unsupervised clustering. Cells were gated on CD45$^+$ alive immune cells after excluding the lymphoid populations.

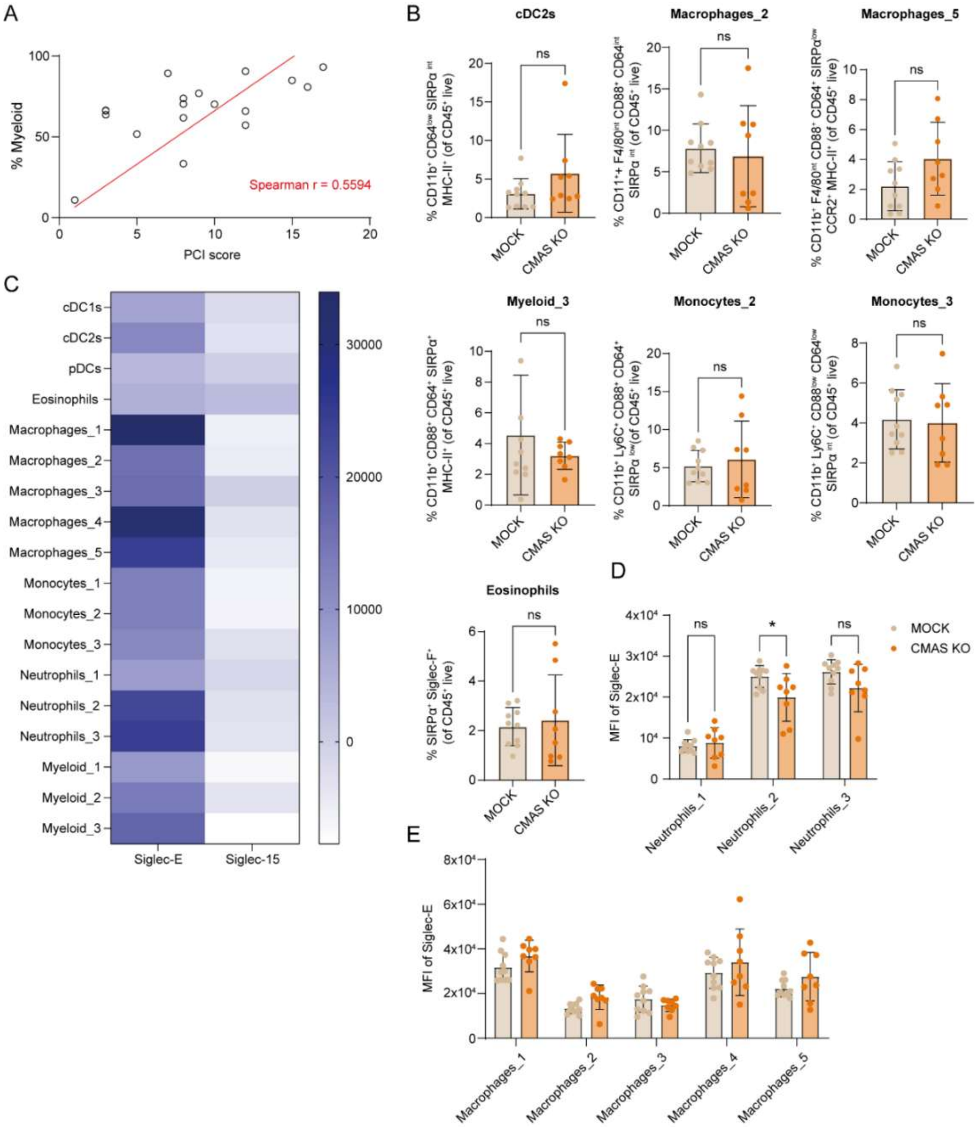

Figure S5. Macrophages and neutrophils exhibit high levels of Siglec-E. (**A**) Spearman correlation plot between the PCI score and the percentage of myeloid cells (CD11b$^+$CD11c$^+$Ly6G$^+$Siglec-F$^+$SIRPα$^+$CD64$^+$F4/80$^+$Ly6C$^+$). (**B**) Percentages of cDC2s, macrophages subsets, a myeloid cluster (CD11b$^+$ Ly6C$^-$F4/80$^-$Ly6G$^-$Siglec-F$^-$), two monocyte subsets and eosinophils in CT26-MOCK and CT26-CMAS KO tumors. Each dot represents one individual mouse. Data is shown as mean ± SD; unpaired non-parametric t-test (ns, not significant). (**C**) Heatmap showing the MFI of Siglec-E and Siglec-15 expression on the different myeloid populations. (**D**) MFI of Siglec-E expression on neutrophils in MOCK and CMAS KO tumors. (**E**) MFI of Siglec-E expression on macrophages in MOCK and CMAS KO tumors. Data is shown as mean ± SD; two-way ANOVA (ns, not significant; *p ≤ 0.05).

A

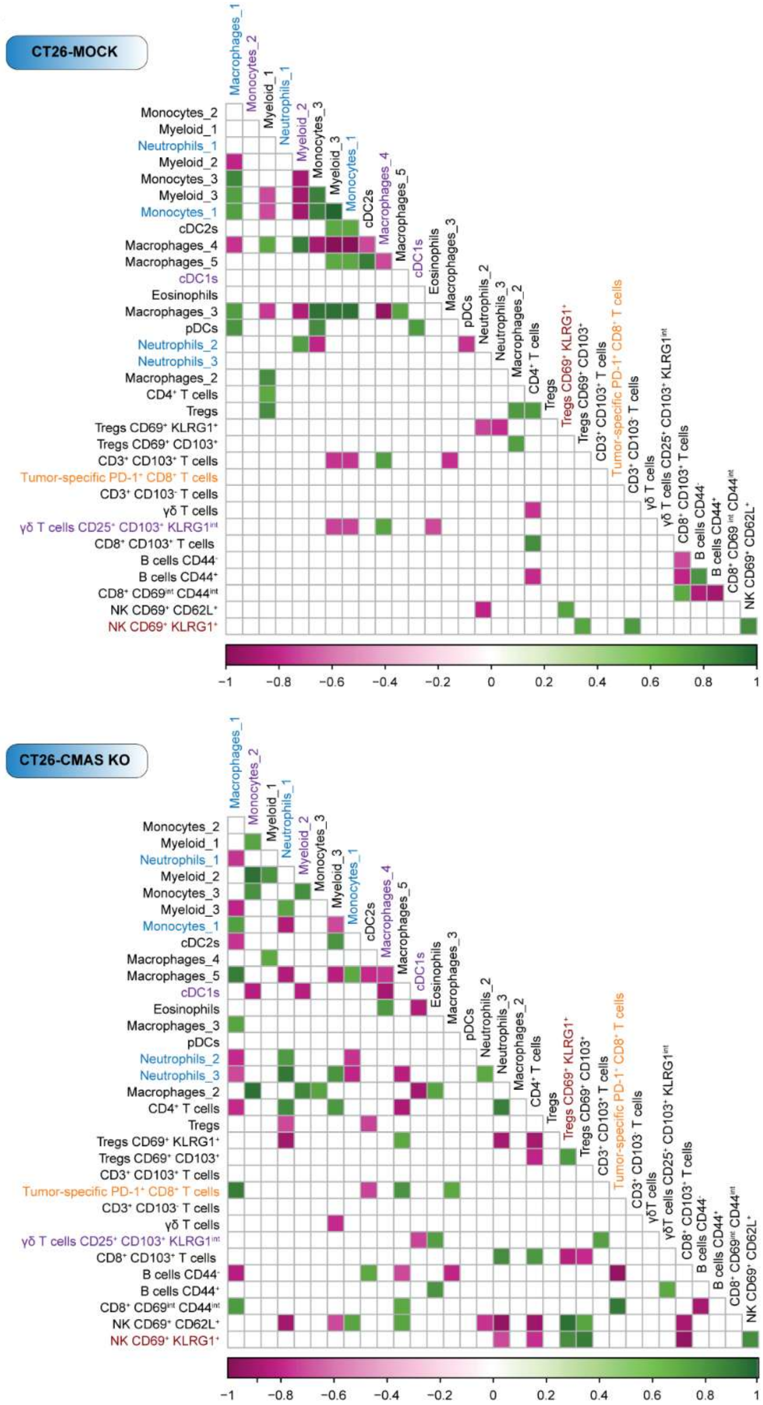

Figure S6. Immune subset correlations in the peritoneal TME. (**A**) Spearman correlation coefficients for all lymphoid and myeloid populations were calculated in R using the corr.test function, and the results were visualized with the corrplot package (colors indicate the matched correlations between immune cell subsets that were only significant in CMAS KO tumors).
